# Hydration-Enhanced Lubricating Electrospun Nanofibrous Membranes Prevent Tissue Adhesion

**DOI:** 10.34133/2020/4907185

**Published:** 2020-03-19

**Authors:** Liang Cheng, Yi Wang, Guoming Sun, Shizhu Wen, Lianfu Deng, Hongyu Zhang, Wenguo Cui

**Affiliations:** ^1^Shanghai Key Laboratory for Prevention and Treatment of Bone and Joint Diseases, Shanghai Institute of Traumatology and Orthopaedics, Ruijin Hospital, Shanghai Jiao Tong University School of Medicine, 197 Ruijin 2nd Road, Shanghai 200025, China; ^2^State Key Laboratory of Tribology, Department of Mechanical Engineering, Tsinghua University, Beijing 100084, China; ^3^College of Chemistry and Environmental Science, Hebei University, Baoding 071002, China; ^4^Affiliated Hospital of Hebei University, Baoding 071000, China

## Abstract

Lubrication is the key to efficient function of human tissues and has significant impact on the comfort level. However, the construction of a lubricating nanofibrous membrane has not been reported as yet, especially using a one-step surface modification method. Here, bioinspired by the superlubrication mechanism of articular cartilage, we successfully construct hydration-enhanced lubricating nanofibers via one-step in situ grafting of a copolymer synthesized by dopamine methacrylamide (DMA) and 2-methacryloyloxyethyl phosphorylcholine (MPC) onto electrospun polycaprolactone (PCL) nanofibers. The zwitterionic MPC structure provides the nanofiber surface with hydration lubrication behavior. The coefficient of friction (COF) of the lubricating nanofibrous membrane decreases significantly and is approximately 65% less than that of pure PCL nanofibers, which are easily worn out under friction regardless of hydration. The lubricating nanofibers, however, show favorable wear-resistance performance. Besides, they possess a strong antiadhesion ability of fibroblasts compared with pure PCL nanofibers. The cell density decreases approximately 9-fold, and the cell area decreases approximately 12 times on day 7. Furthermore, the in vivo antitendon adhesion data reveals that the lubricating nanofiber group has a significantly lower adhesion score and a better antitissue adhesion. Altogether, our developed hydration-enhanced lubricating nanofibers show promising applications in the biomedical field such as antiadhesive membranes.

## 1. Introduction

Electrospinning is a robust technology to fabricate functional nanofibers [1]. Manipulating the composition, structure, and surface property enabled researchers to develop electrospun nanofibers with unique performances such as superhydrophobicity/hydrophilicity [2–4], piezoelectric conversion [5, 6], and multiple response [7, 8]. Electrospun nanofibers have found extensive applications in the energy, environment, and biomedical field [1]. In biomedical applications, adjusting the surface properties of the nanofibers (e.g., fiber orientation and patterned structure) is frequently employed to achieve specific cell adhesion and growth on fiber surfaces [9, 10]. Favorable cell adhesion is the key to tissue regeneration [11], but cell adhesion could lead to serious undesirable consequences under certain circumstances, such as tendon adhesion [12], intestinal adhesion [13], and intrauterine adhesion [14]. Therefore, developing electrospun nanofibrous membranes (ENMs) that can completely inhibit tissue adhesion may open up novel functional applications.

The encapsulation of drugs such as mitomycin and ibuprofen within electrospun nanofibers is commonly used to inhibit cell adhesion on the surface of ENMs, but the local side effects of the drugs restrict their clinical applications [15–17]. Accordingly, it is very desirable to develop electrospun nanofibers without any drugs to achieve effective antiadhesive performance. Notably, it has been reported that the surface hydration lubrication properties of the materials are related to the bioadhesion [18, 19]. The hydration layer on a parylene film is highly effective for preventing cell adhesion [18]. Moreover, the lubricated surface can reduce wear caused by tissue sliding. Collectively, developing hydration-enhanced lubricating ENMs may achieve complete inhibition of cell adhesion on the nanofiber surfaces.

Hydration lubrication is an effective approach to achieve superlubrication [20]. Jahn et al. [21] revealed that the presence of a stable hydration layer contributed to the ultralow coefficient of friction (COF) between articular cartilages. The phosphatidylcholine lipid in human articular cartilage has a zwitterionic structure, where the positively charged (N+(CH3)3) group and the negatively charged (PO4−) group can strongly adsorb water molecules to form a stable hydrated lubrication layer. This strongly bounded water layer can separate the two friction surfaces all the time, thus enabling the ultralow COF between articular cartilages [21]. A stable hydrated lubrication layer can not only lead to ultralow COF between two surfaces under shear but also easily detach adhesive proteins from the material surface [22]. On these highly lubricated surfaces, it is very difficult for the cellular pseudopods to form a strong focal adhesion; thereby, complete inhibition of cell adhesion can be achieved. Therefore, we hypothesized that the integration of hydration-enhanced lubricating surfaces onto electrospun nanofibers will allow the ENMs to completely inhibit cell adhesion.

Currently, 2-methacryloyloxyethyl phosphorylcholine (MPC) that has a positively charged (N^+^(CH3)_3_) group and a negatively charged (PO4^−^) group is a commonly used zwitterionic material with excellent biocompatibility [23, 24]. The typical method to incorporate a hydrated surface is to graft pMPC polymer brushes onto the material surface [25, 26]. However, chemical grafting methods, such as atom transfer radical polymerization (ATRP) and reversible addition-fragmentation chain transfer polymerization (RAFT) [27, 28], often involve complicated chemical synthesis that utilizes toxic chemicals and organic solvents, which can damage the nanofiber structure [29]. Mussel byssus has abundant dopamine-based molecules that enable it with strong self-adhesion ability [30]. Our prior studies showed that the mussel-inspired dopamine-based one-step grafting allows us to incorporate functional molecules to the nanofiber surface [31, 32]. Therefore, in this work, we successfully developed hydration-enhanced lubricating ENMs through one-step grafting of pMPC molecules onto electrospun polycaprolactone (PCL) nanofiber surface following our bioinspired catecholamine chemistry-based grafting strategy, and the effectiveness of anticell adhesion was also evaluated. Firstly, we synthesized poly (DMA-co-MPC) molecules by free radical copolymerization of dopamine methacrylamide (DMA) and MPC and then successfully one-step grafted the copolymer onto the surface of the electrospun PCL nanofibrous membrane by a dip-coating method. The friction and wear experiments were carried out to examine the hydration lubrication effect. We evaluated the adhesion of fibroblasts in vitro on the nanofibrous membrane surface. To determine the potential biomedical applications as antiadhesive biomembranes, we further implanted the developed ENMs into a rat tendon adhesion model and monitored the in vivo effect.

## 2. Results

### 2.1. One-Step Grafting of pMPC onto Electrospun Nanofibers

**Figure 1:**
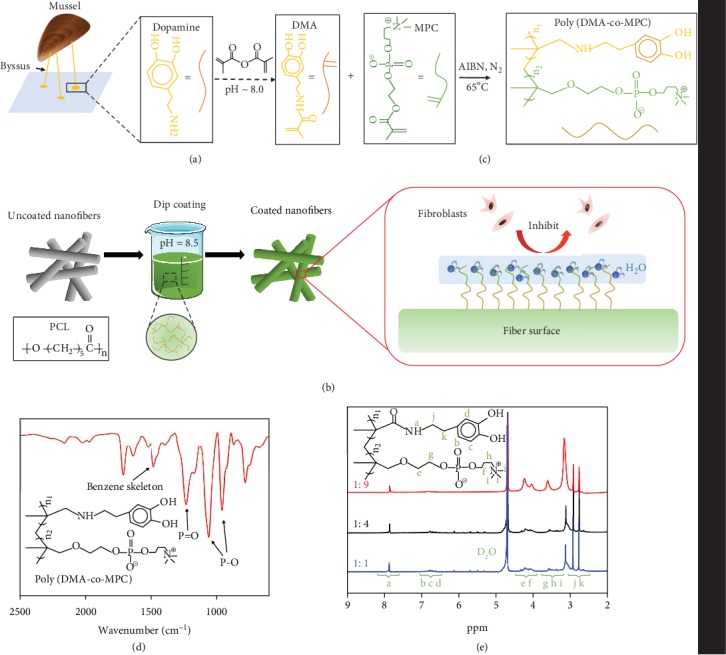
Illustration of the one-step surface grafting process. (a) Synthesis process of poly (DMA-co-MPC) and (b) the dip-coating method to graft pMPC onto the PCL fibrous membrane surface. (c) The coated membrane surface is expected to form a stable hydration layer to inhibit cell adhesion. (d) FTIR spectrum and (e) ^1^H NMR spectrum of poly (DMA-co-MPC).

We used a mussel-inspired dopamine-based approach to functionalize the PCL nanofiber surface. Carbon double bonds were first incorporated into DMA by reacting methacrylic anhydride with dopamine (Figure 1(a)). Free radical polymerization was then carried out to synthesize poly (DMA-co-MPC) with different raw ratios of DMA and MPC at 1 : 1, 1 : 4, and 1 : 9 (Figure 1(a)), respectively. The FTIR spectrum of poly (DMA-co-MPC) preliminarily confirmed the successful preparation of the copolymer (Figure 1(d)), evidenced by the corresponding peaks of benzene skeleton (1400-1600 cm^−1^), P-O and P=O groups (960 cm^−1^, 1060 cm^−1^, and 1244 cm^−1^). The ^1^H nuclear magnetic resonance (NMR) spectrum further indicated that poly (DMA-co-MPC) was successfully synthesized (Figure 1(e)), and the pMPC concentration could be adjusted by the raw ratio of DMA versus MPC. After poly (DMA-co-MPC) products were synthesized, the dip-coating method was used to achieve one-step grafting of pMPC onto electrospun PCL nanofibers (Figure 1(b)). Additional FTIR results revealed that all membranes are successfully coated with poly (DMA-co-MPC) (Fig. [Supplementary-material supplementary-material-1]), regardless of their ratios. Chen et al. [22] reported that the zwitterionic structure of pMPC was capable of strong adsorption of water molecules; we thus hypothesized that the pMPC-grafted nanofiber surface could form a stable hydrated layer that would further prevent cell adhesion (Figure 1(c)).

**Figure 2:**
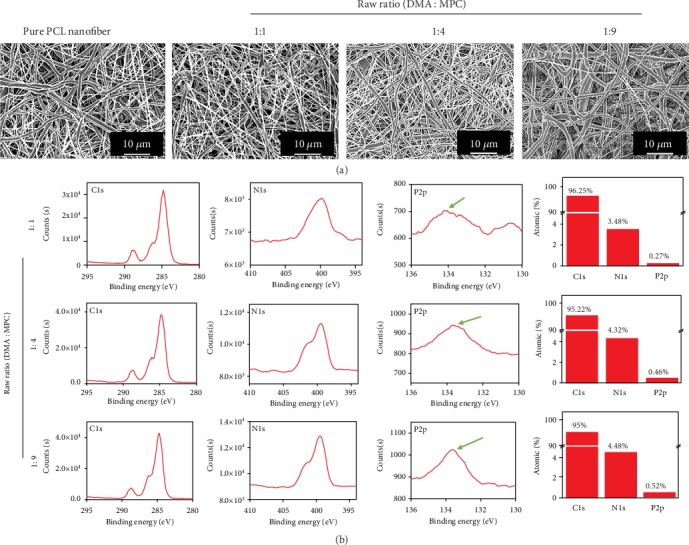
Characterizations of electrospun nanofibers. (a) SEM images of the electrospun PCL fibrous membrane and pMPC-grafted membranes with different raw ratios. (b) XPS results of different pMPC-grafted membranes.

The pMPC-grafted nanofibrous membranes were further confirmed with SEM and XPS results (Figure 2). All nanofibers showed a relatively uniform porous nanofibrous structure (Figure 2(a)), indicating that the bioinspired one-step surface grafting process caused no damage to the fiber structures. The typical peaks of N and P elements were found in all membranes from the XPS results, which suggested that pMPC had been successfully grafted onto the surfaces of the nanofiber membranes (Figure 2(b)), as N and P elements only came from pMPC. Moreover, the percentage of N and P atoms increased with the increase of MPC contents in poly (DMA-co-MPC), which enabled us to regulate the amounts of pMPC grafted on the fiber surfaces by adjusting the raw ratios of DMA versus MPC.

### 2.2. Lubrication Performance

**Figure 3:**
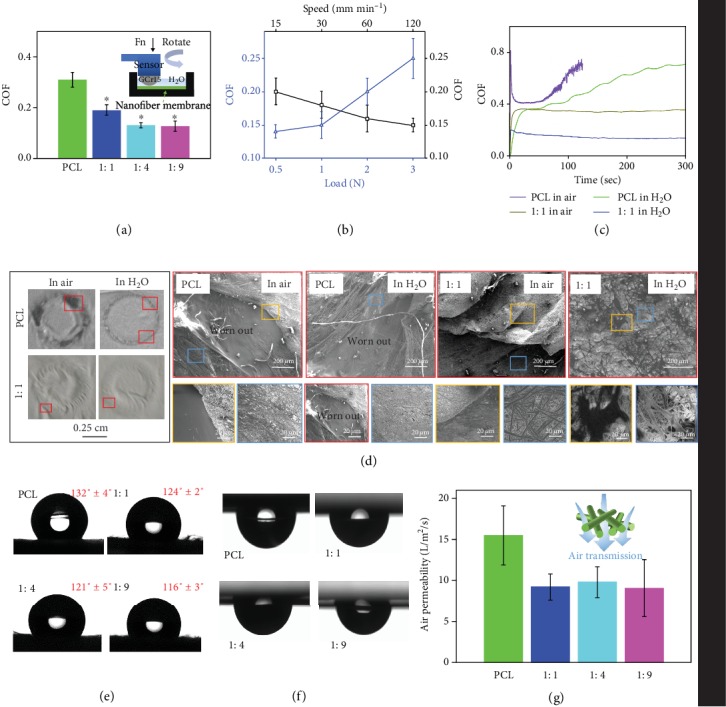
Lubrication tests for the different nanofibrous samples. (a) COF of all the samples. Inset is the schematic diagram of the rotation mode. (b) COF under different normal loads and rotation speeds. (c) Time-COF curves for PCL and 1 : 1 membranes tested in air and in water. (d) Photos and SEM images of wear conditions after being tested in air and in water for PCL and 1 : 1 membranes. The magnified SEM images with different color borders are corresponding to sites with different colors. (e) Water contact angles of all the membrane samples. (f) Water droplets were vertically inverted and adsorbed on the sample surfaces. (g) Air permeability of all the fibrous samples.

Given polymeric nanofibers were soft materials that were easily broken under reciprocating friction modes, we used rotation mode at constant speed to determine the lubrication performance of the ENMs (Figure 3(a)). Electrospun PCL nanofibrous membranes had a relatively high COF at around 0.32, while 1 : 1, 1 : 4, and 1 : 9 membranes possessed significantly lower COF at approximately 0.19, 0.14, and 0.10, respectively (Figure 3(a)). Our results clearly demonstrated that the pMPC-grafted ENMs had remarkable lubrication properties, and the COF of membrane surface decreased with the increase of pMPC content. Both normal loads and rotation speeds influenced the COF (Figure 3(b)). When the rotation speed increased from 15 mm min^−1^ to 120 mm min^−1^, the COF reduced from around 0.2 to 0.15. This decrease might be attributed to the deformations under different applied loads [33, 34]. The higher load would induce larger elastic deformation and more resistance, resulting in the increased friction forces. In addition, the increased rotation speed would lead to decreased contact time, resulting in smaller elastic deformation which would induce lower friction forces.

To examine the hydration lubrication effect, PCL and 1 : 1 membranes were measured for lubrication testes in both water and air, respectively. The COF of the PCL sample increased dramatically within 50 s in both water and air due to the lack of surface lubrication (Figure 3(c)). The COFs of the membrane, however, stayed stable once they reached the plateau, and the COF in water was almost 2-fold less than that in air. Our results clearly demonstrated that integration of the pMPC onto the fiber surface induced hydration lubrication of the nanofibers, and a stable hydration layer could be formed in the presence of a large amount of water molecules [20]. SEM images after the friction tests indicated that PCL sample was worn out more severely in water than in air (Figure 3(d)). This result illustrated that water alone had no lubricant effect on PCL membranes. However, no macroscopic worn areas were observed for 1 : 1 membrane tested in either water or air. Though noticeable wrinkles were observed on the 1 : 1 membrane, no nanofiber structures were destroyed, and an almost fully intact structure remained in water. This was because a more stable hydration layer which was formed around the zwitterionic charges of the phosphorylcholine group could form from aqueous water than from vapor water in air. The water molecule had a large electric dipole due to the residual charges on the H and O atoms and therefore could attach onto the zwitterionic charges based on water dipole−charge interaction. These water molecules (12–19 per headgroup) could support large pressure and respond in a fluid-like manner under shear, resulting in greatly reduced COF (and thus enhanced lubrication) at the interface [20, 35]. In light of aforementioned results, we can conclude that our pMPC-grafted nanofibers possess greater wear-resistance property than pure PCL nanofibers due to the hydration lubrication mechanism.

### 2.3. Characterizations of Electrospun Nanofibers

The water contact angles, air permeability, and tensile strength properties were measured for all membranes. The water contact angles of PCL, 1 : 1, 1 : 4, and 1 : 9 membranes were 132° ± 4, 124° ± 2, 121° ± 5, and 116° ± 3 (Figure 3(e)), respectively. All sample surfaces were hydrophobic as the water contact angles were all above 90°. Owing to the hydration lubrication effects of MPC molecules, the water contact angles decreased with the increase of MPC molecules on fiber surface. Maeda et al. reported that electrospun hydrophilic pMPC nanofibers had a water contact angle at approximately about 120°, in which the porous nanofibrous structure contributed to hydrophobic surfaces [36]. Additionally, all the fiber sample surfaces were able to hold the water drops after we turned them upside down (Figure 3(f)), and the droplets were smaller in pMPC-grafted membranes. This indicated that the developed membranes were able to retain water, which could make the sample better fit the surrounding tissue and maintain nutrient adsorption when implanted in vivo. All the pMPC-grafted membranes possessed optimal air permeability since the air permeability of 1 : 1, 1 : 4, and 1 : 9 membranes was between 5 and 15 L m^−2^ s^−1^, though it was a little lower than that of the PCL membrane (Figure 3(g)). There was no significant difference between PCL and pMPC-grafted nanofibers for Young's modulus, break stress, and elongation at break (Fig. [Supplementary-material supplementary-material-1]). This revealed that the one-step dip-coating method did not affect the mechanical properties. Collectively, our results demonstrated that the pMPC-grafted membranes were promising candidates of implantable biomembranes, especially as antitissue adhesion membranes.

### 2.4. In Vitro Anticell Adhesion

**Figure 4:**
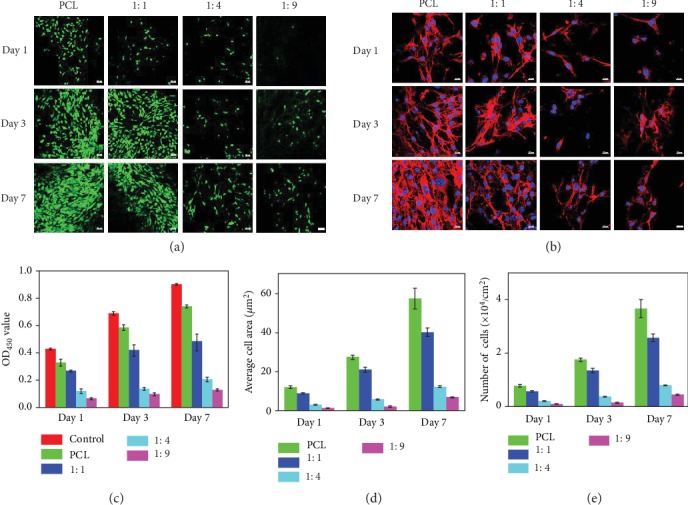
In vitro antiadhesion results. (a) Live/Dead staining CLSM images (scale bar: 50 *μ*m) and (b) cytoskeleton staining images (scale bar: 20 *μ*m) on days 1, 3, and 7 of fibroblasts cultured on the different nanofiber surfaces. (c) OD value, (d) average cell area, and (e) number of cells on days 1, 3, and 7.

After being cultured for 1, 3, and 7 days, the fibroblasts seeded on the surface of ENMs (PCL, 1 : 1, 1 : 4, and 1 : 9) were evaluated for proliferation, viability, and adhesion. In the Live/Dead staining images on days 1, 3, and 7 (Figure 4(a)), only green fluorescence that represents live cells was found on all the sample surfaces, indicating all the membranes showed good biocompatibility. However, the cell density decreased gradually as the amount of pMPC increased on the membrane surface (Figure 4(a)). Moreover, the optical density (OD) values (Figure 4(c)) and cell numbers (Figure 4(d)) further showed consistent results with the cell viability test. In the meantime, the cell growth rates were lower in the 1 : 1, 1 : 4, and 1 : 9 membrane groups than those in the PCL membrane group, which indicates that pMPC-grafted membranes were able to reduce the number of cell attachment.

The cytoskeletal dyes consisted of F-actin protein (red, cytoskeletal), and the nucleus (blue, cell nucleus) were used to investigate cell spreading on different membrane surfaces (Figure 4(b)). The staining results (Figures 4(b) and 4(e)) revealed that cells on PCL membrane surface spread well and had the largest cell area; however, cells on other pMPC-grafted membranes presented cytoplasmic shrinkage and substantially reduced cell areas. In addition, with the increase of pMPC grafting yield, the flattened morphology of cells on the surfaces reduced gradually as the same decreased tendency of the cell areas. Cell areas on the surfaces of 1 : 4 and 1 : 9 membrane had no significant increase on day 7, suggesting that these fibers have long-lasting antiadhesion ability. Clearly, zwitterionic pMPC molecules were grafted onto the fiber surfaces and formed hydration lubrication layer, which greatly inhibited cell spreading. Taken together, our pMPC-grafted nanofibers could form stable hydration lubrication layer and have strong anticell adhesion ability.

### 2.5. In Vivo Antiadhesive Performance

**Figure 5:**
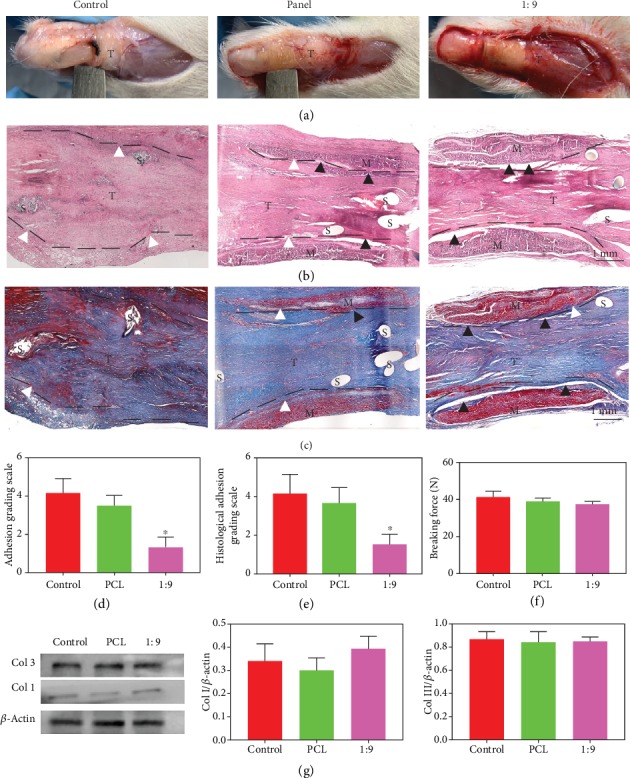
In vivo experiment performance. (a) Photos of operated limbs after 21 days. (b) H&E staining and (c) Masson staining images of the harvested tissues. T: tendon; M: membranes; S: suture. (d) Adhesion grading scale, (e) histological adhesion grading scale of control, PCL, and 1 : 9 membrane groups. (f) Breaking forces of the repaired tendon of control, PCL, and 1 : 9 membrane groups. (g) Western blot data for each group. ^∗^*p* < 0.05 (compared with the control group).

After 21 days of treatments, the tendon adhesions around implanted zones were initially evaluated by direct observation (Figure 5(a)). No obvious signs of inflammation or ulcer around the incision were discovered. In the untreated control group, large and severe peritendinous adhesion areas could be observed at the repair site, while the knife handle could hardly pass the space between the sutured tendon and the surrounding tissue. The experimental groups however showed completely opposite results. Under the treatment of PCL membranes, only small bundles of muscle fibers bridged to the surrounding tissues. It was interesting to find the antiadhesion effect was pronounced in the 1 : 9 membrane group. Little adhesion was observed between the peritendinous tissue and the repaired tendon, which enabled the flexible laterally movement of the handle. Based on these surgical findings, the control group should be given the highest scores of mainly 4 and 5, the PCL group should be scored for mainly 3 and 4, while the 1 : 9 membrane group should be given scores 1 and 2. The statistical result of scores for the 1 : 9 membrane group was significantly lower than that of PCL group (Figure 5(d)), revealing that 1 : 9 membrane may possess strong antiadhesive capability. Typical histological sections of the repaired tendons in each group showed the treatment results (Figures 5(b) and 5(c)). A mass of fibrous tissues adulterated the tissue around the site of repaired tendon in the control group. In the PCL group, loose bundles of fibrous tissues could be found around the repaired tendon, while in the 1 : 9 membrane group, only little scattered peritendinous adhesions were observed. This indicated that the 1 : 9 membrane repelled the fibroblast deposition and exhibited great potential as the optimum repair material. In the control group, the muscle fiber bundles were disorderly arranged and grew into the laceration site along with adhesion tissues, resulting in undesirable tendon healing. In contrast, in the PCL and 1 : 9 groups, fiber bundles were temperately aligned, and the repaired tendon was well connected at the broken ends. The histological adhesion grade of the 1 : 9 group is significantly lower than that of the PCL group (Figure 5(e)). Additionally, the breaking forces that represented the recovery outcomes of the repaired tendon showed no significant difference among all the groups in the mechanical evaluation results (Figure 5(f)). Altogether, our in vivo antiadhesive measurements demonstrated 1 : 9 membranes had excellent antitissue adhesion potential.

We further evaluated the regeneration-related proteins including collagen type I and type III through western blot methods. Collagen I expression in the 1 : 9 membrane group was slightly higher than that of the control group and the PCL group (Figure 5(g)), but no significant difference was observed among the three groups. In the meantime, no significant difference was found among the three groups for collagen III expression. All these data suggested that the 1 : 9 membrane has antiadhesion ability. Though it could not promote the healing of tendon performance, the 1 : 9 membrane did not inhibit the tendon healing process. The tendon healing system involves intrinsic healing and extrinsic healing, while the extrinsic healing might lead to the formation of tendon adhesion with the character of numerous exogenous fibroblast invasion and excessive extracellular matrix synthesis. In our research, the 1 : 9 membrane achieves excellent biological barrier function, while does not affect the intrinsic healing process.

## 3. Discussion

In this study, we had developed novel hydration-enhanced lubricating ENMs through bioinspired one-step surface functionalization and explored the potential biomedical applications such as antiadhesive biomembranes. Based on the catecholamine chemistry grafting strategy, zwitterionic pMPC molecules were one-step grafted onto the surface of electrospun nanofibers with different concentrations. The lubrication tests showed that the pMPC-grafted nanofibrous membrane exhibited enhanced lubrication property, thus presenting favorable wear-resistance performance. Besides, the developed membranes were breathable enough to allow nutrient exchange. Moreover, the lubricating membranes had excellent anticell adhesion characteristics. Furthermore, the results of in vivo antitendon adhesion experiment demonstrated that the lubricating nanofiber membranes could be an optimal candidate as antiadhesive biomembranes. We believe that this novel design strategy for lubricating nanofiber membranes will be highly promising for clinic applications.

## 4. Materials and Methods

### 4.1. Synthesis of Poly (DMA-co-MPC)

DMA was prepared based on the previous literature [37]. Firstly, 5 g dopamine hydrochloride, 10 g sodium borate, and 4 g sodium bicarbonate were codissolved in 100 mL deionized (DI) water under nitrogen atmosphere (N_2_). Then, the solution was added with 5 mL methacrylic anhydride and 25 mL tetrahydrofuran, and 0.2 M NaOH solution was added to adjust the pH of the above solution to about 8. After being stirred for 12 h under N_2_, the reacted solution was adjusted to pH < 2 using 0.5 M hydrochloric acid, then purged and extracted with ethyl acetate, and filtered with excessive magnesium sulfate. Eventually, DMA products could be obtained by precipitating the filtered solution with n-hexane.

Poly (DMA-co-MPC) was synthesized based on the free radical copolymerization of DMA and MPC by using AIBN (2, 2-azodiisobutyronitrile, C_8_H_12_N_4_) as the initiator. Briefly, a total weight of 1 g DMA and MPC with different raw ratios (1 : 1, 1 : 4, and 1 : 9) was codissolved with 3 mg AIBN in 50 mL DMF (N,N-dimethylformamide), followed by stirring for 24 h at 65°C under N_2_. After the reaction finished, the final solution was dialyzed against DI water and then freeze dried for 3 days to obtain poly (DMA-co-MPC) product.

### 4.2. One-Step Grafting Process

Electrospun PCL nanofibers were prepared based on our previous studies [38–40]. Briefly, 2 g PCL was dissolved in 20 mL trifluoroethanol and then electrospun into nanofibrous membrane following the parameters of 20 kV voltage, 6 mL h^−1^ feeding speed, and 18 cm distance between a needle tip and a collector. The dip-coating method was used to achieve one-step grafting of pMPC onto PCL nanofiber surfaces. Poly (DMA-co-MPC) with different raw ratios were dissolved in Tris-HCL solution (pH~8.5) as a concentration of 1 mg mL^−1^. Then, electrospun PCL nanofibrous membranes were soaked in the poly (DMA-co-MPC) solution for 24 h. After that, membranes were taken out and washed thrice with DI water to remove physically adsorbed polymers on the surface. The final obtained ENMs from different DMA/MPC raw ratios were labelled as 1 : 1 membrane, 1 : 4 membrane, and 1 : 9 membrane, respectively.

### 4.3. Characterizations

For FTIR (Fourier transform-infrared spectroscopy) measurements, samples were tested through a Nicolet 6700 infrared spectrometer (Thermo Electron Corporation, Shanghai, China). ^1^H HMR spectra of poly (DMA-co-MPC) with different raw ratios were recorded via a nuclear magnetic resonance spectrometer (AVANCE III HD 400 MHz, Bruker, Switzerland). For morphology observations, all the samples were first sprayed with Pt for 10 min through a vacuum ion sputtering apparatus (EM ACE600, Leica, Germany) and then scanned at random views via a scanning electron microscope (SEM, Quanta200, FEI, Eindhoven, Netherlands). XPS (X-ray photoelectron spectroscopy) results were obtained via a 250XI X-ray photoelectron spectroscopy instrument (Thermo, Waltham, USA). Water contact angles were measured using an OCA-20 contact angle system (DataPhysics Instruments, Filderstadt, Germany). Mechanical properties of the nanofiber samples (5 cm (length) × 6 mm (width)) were conducted through a mechanical testing machine (Instron 5567, Norwood, MA), as we previously reported [38, 40]. The air permeability of the nanofibrous samples was determined based on EN ISO 9237 standard via an air permeability tester (FX 3300, TEXTEST AG Zurich, Switzerland) under 100 Pa air pressure.

### 4.4. Lubrication Tests

The lubrication tests were performed using a universal materials tester (UMT-5, Bruker Nano Inc., Germany) under rotation modes (rotation speed 50 mm min^−1^, normal load 0.5 N, rotation radius 3 mm, DI water). As shown in Figure 3(a), the ENMs as the lower specimens were flatly bonded to the surface of glass slides using 3M tapes, while GCr15 steel balls with 6 mm diameter were used as the upper specimens. The 1 : 1 sample group was chosen to evaluate the influence of different experimental conditions on the tribological performance, including normal load (0.5-3 N) and rotation speed (15-120 mm min^−1^). PCL and 1 : 1 samples were measured for COF with or without DI water (in air or in water) to confirm the hydration lubrication effect. After the lubrication tests finished, naturally dried PCL and 1 : 1 samples were recorded for photos to observe the worn conditions.

### 4.5. Cell Experiments

All the nanofiber samples were sterilized prior to the cell experiments. Briefly, nanofiber samples were placed into 24-well culture plates and then sterilized in 75% ethanol aqueous solution for 10 min, as well as soaked in PBS supplied with 1% penicillin streptomycin for another 15 min. Finally, the dried samples were sterilized under UV irradiation for another 3 h.

NIH/3T3 fibroblasts were used to assess the cell growth on the surface of fibrous membranes [41]. The Dulbecco's modified Eagle's medium (DMEM, 11885084, Gibco, US) culture media supplied with 10% fetal bovine serum (FBS, 10100154, Gibco, US) and 1% penicillin-streptomycin (PS, SV30010, HyClone, US) were used and refreshed every two days. The ENMs were cut into disc shapes with a diameter of 15 mm and then placed into 24-well plates followed by sterilization under UV light for 24 h. Subsequently, suspensions of cells with a density of 4 × 10^4^ per well were seeded onto the different ENM surfaces and incubated under 5% CO_2_ at 37°C. Cell Counting Kit-8 (CCK-8, ck04, Dojindo, Japan) and Live/Dead Cell Kit (Invitrogen, L3224, US) were used to evaluate cell proliferation after cultured for 1, 3, and 7 days. The detailed procedures were according to the kit protocols. For CCK-8 measurements, absorbance at 450 nm of each sample was recorded through a microplate reader (Infinite F50, TECAN, Switzerland). For observation of Live/Dead staining, a confocal laser scanning microscope (CLSM, LSM800, ZEISS, Germany) was employed. Besides, fluorescent staining of the cytoskeletal arrangements on fibrous membranes using phalloidin (Invitrogen, A12379, US) and DAPI (Sigma, S7113, US) was also conducted. The staining steps were referred to our previous work [17]. CLSM was also employed to obtain the cytoskeletal-stained images on 1, 3, and 7 days. The average cell densities and average cell areas were counted by using ImageJ software from the CLSM images.

### 4.6. Animal Experiments

All the animal experiments were approved by the Animal Care and Use Committee of Shanghai Jiao Tong University, School of Medicine, and all the operation procedures were performed in accordance with the National Institutes of Health Guide for Care. The sterilization process of the nanofiber membranes was the same to that in Section 4.5. The surgical operation process was shown in Fig. [Supplementary-material supplementary-material-1]. Eighteen male SD rats, weighting between 200 g and 250 g, received intraperitoneal anesthesia of pentobarbital sodium (30 mg kg^−1^). Afterwards, their limbs were shaved and sterilized before a lateral incision was made. The tendon was transected at the site of 5 mm away from the calcaneal tuberosity and repaired immediately with 4-0 silk suture via a modified Kessler suture technique (Ethicon Ltd., Edinburgh, UK), as reported in our previous studies [17, 42]. Then, the prepared membranes were used to wrap the surgical site, with the lubricating surface of the membranes facing to the surrounding tissue. Three groups were set up as follows: control group with no treatment, experiment groups with wrapping of PCL or 1 : 9 membranes.

### 4.7. Macroscopic Observation and Analysis

On 21 days after the operations, images of the operated limbs were taken before sacrificing the rats to examine any inflammation or ulcer. Besides, according to the surgical findings, the severity of peritendinous adhesion in a particular area was quantified in the form of adhesion scores from 1 to 5. The definition of adhesion scores was based on our previous studies [42, 43]: score 1 means nearly no adhesions; score 2 means filmy adhesions easily separable by blunt dissection; score 3 means less than or equal to 50% of the adhesion areas must be separated by sharp dissection; score 4 means 51-97.5% of the adhesion areas must be separated by sharp dissection; score 5 means more than 97.5% of the adhesion areas must be separated by sharp dissection.

### 4.8. Histological Staining Analysis

The dissected tendons on the repaired site were fixed in 4% paraformaldehyde for 24 h. Then, these tendons were embedded into paraffin, cut into sections of 5 *μ*m thickness, dehydrated through a graded series of ethanol, and stained with hematoxylin and eosin (H&E) and Masson's trichrome. Photos of histologic adhesion areas were taken by using light microscopy (Axio CSM700, Zeiss, Germany). Similarly, histologic adhesions were also quantified into five grades as follows: grade 1, no adhesions; grade 2, mild (<25% adhesion area of the tendon surface); grade 3, moderate (25-50% adhesion area of the tendon surface); grade 4, severe (50-75% adhesion area of the tendon surface); and grade 5, most severe (>75% adhesion area of the tendon surface).

### 4.9. Biomechanical Evaluation

The recovery situation of the repaired tendon was evaluated by a rheometer (ElectroForce DMA3200, TA Instruments, USA) via measuring the maximal breaking force. After the both sides of the tendon were fixed in the rheometer, the tendon ends were pulled apart at a speed of 10 mm min^−1^ until the tendon was ruptured and the rheometer recorded the maximal breaking force.

### 4.10. Western Blot Analysis

The repaired tendon sites in different groups were collected, ground using a high-throughput tissue grinder, immersed in a tube filled with liquid nitrogen, and then homogenized in 980 *μ*L ice-cold RIPA (P0013D, Beyotime, China) supplemented with 10 *μ*L PMSF (ST506, Beyotime, China) and 10 *μ*L protease inhibitor (P1006, Beyotime, China), respectively. The mixture was incubated on ice for 30 min and then centrifuged at 12,000 rpm for 10 min. The supernatant was collected, and the protein concentrations were measured by the BCA protein assay kit (P0012S, Beyotime, China). The samples were subjected to 10% SDS-PAGE gel electrophoresis and then transferred to a PVDF membrane (Millipore, USA). After blocking with 5% skim milk, the membranes were incubated with antibodies against collagen I and III (ab34710, ab7778, Abcam, USA) and *β*-actin (ab179467, Abcam, USA) overnight at 4°C. After washing with TBST, the membranes were incubated with the HRP-conjugated secondary antibodies (ab205718, Abcam, USA) for 1 h at room temperature. The antigen–antibody complexes were visualized by using an enhanced chemiluminescence detection system as recommended by the manufacturer. The signal intensity was quantified using ImageJ software.

### 4.11. Statistical Analysis

All data were presented as mean ± standard deviation. One-way analysis of variance was performed to assess statistical difference using SPSS 19.0 software. Statistical significance was considered when ^∗^*p* < 0.05.

## Supplementary Materials

Supplementary Materials Figure S1: additional FTIR results of poly (DMA-co-MPC), 1 : 1, 1 : 4, and 1 : 9 membranes. Figure S2: mechanical properties of all membranes. Figure S3: operation process of rat Achilles tendon antiadhesion.
