# Supplementary material for: Hydration-Enhanced Lubricating Electrospun Nanofibrous Membranes Prevent Tissue Adhesion
Source: Research (Wash D C). 2020 Mar 19;2020:4907185. doi: 10.34133/2020/4907185 (PMC7106391; doi:10.34133/2020/4907185)
Supplement: Supplementary Materials — Figure S1: additional FTIR results of poly (DMA-co-MPC), 1 : 1, 1 : 4, and 1 : 9 membranes. Figure S2: mechanical properties of all membranes. Figure S3: operation process of rat Achilles tendon antiadhesion. [file 4907185.f1.docx]

Supporting Information

**Hydration-Enhanced Lubricating Electrospun Nanofibrous Membranes Prevent Tissue Adhesion**

Liang Cheng, Yi Wang, Guoming Sun, Shizhu Wen, Lianfu Deng*, Hongyu Zhang*, and Wenguo Cui*

^1^Shanghai Key Laboratory for Prevention and Treatment of Bone and Joint Diseases, Shanghai Institute of Traumatology and Orthopaedics, Ruijin Hospital, Shanghai Jiao Tong University School of Medicine, Shanghai 200025, P. R. China.

^2^State Key Laboratory of Tribology, Department of Mechanical Engineering, Tsinghua University, Beijing 100084, P. R. China.

^3^College of Chemistry and Environmental Science, Hebei University, Baoding 071002, P. R. China.

^4^Affiliated Hospital of Hebei University, Baoding 071000, P. R. China .

Correspondence should be addressed to Lianfu Deng: lf_deng@126.com; Hongyu Zhang: [zhanghyu@mail.tsinghua.edu.cn](mailto:zhanghyu@mail.tsinghua.edu.cn); Wenguo Cui: [wgcui80@hotmail.com](mailto:wgcui80@hotmail.com).

†: These authors contributed equally to this work.

**Supplemental Materials and Methods**


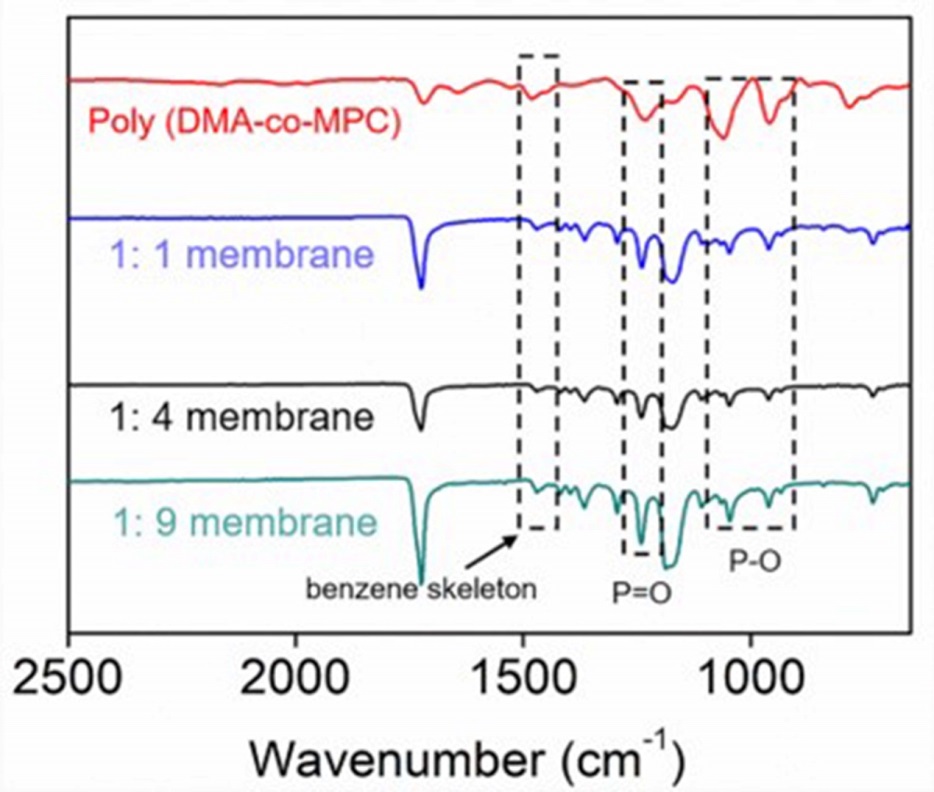


**Figure S1.** Additional FTIR results of poly (DMA-co-MPC), 1:1, 1:4 and 1:9 membranes.


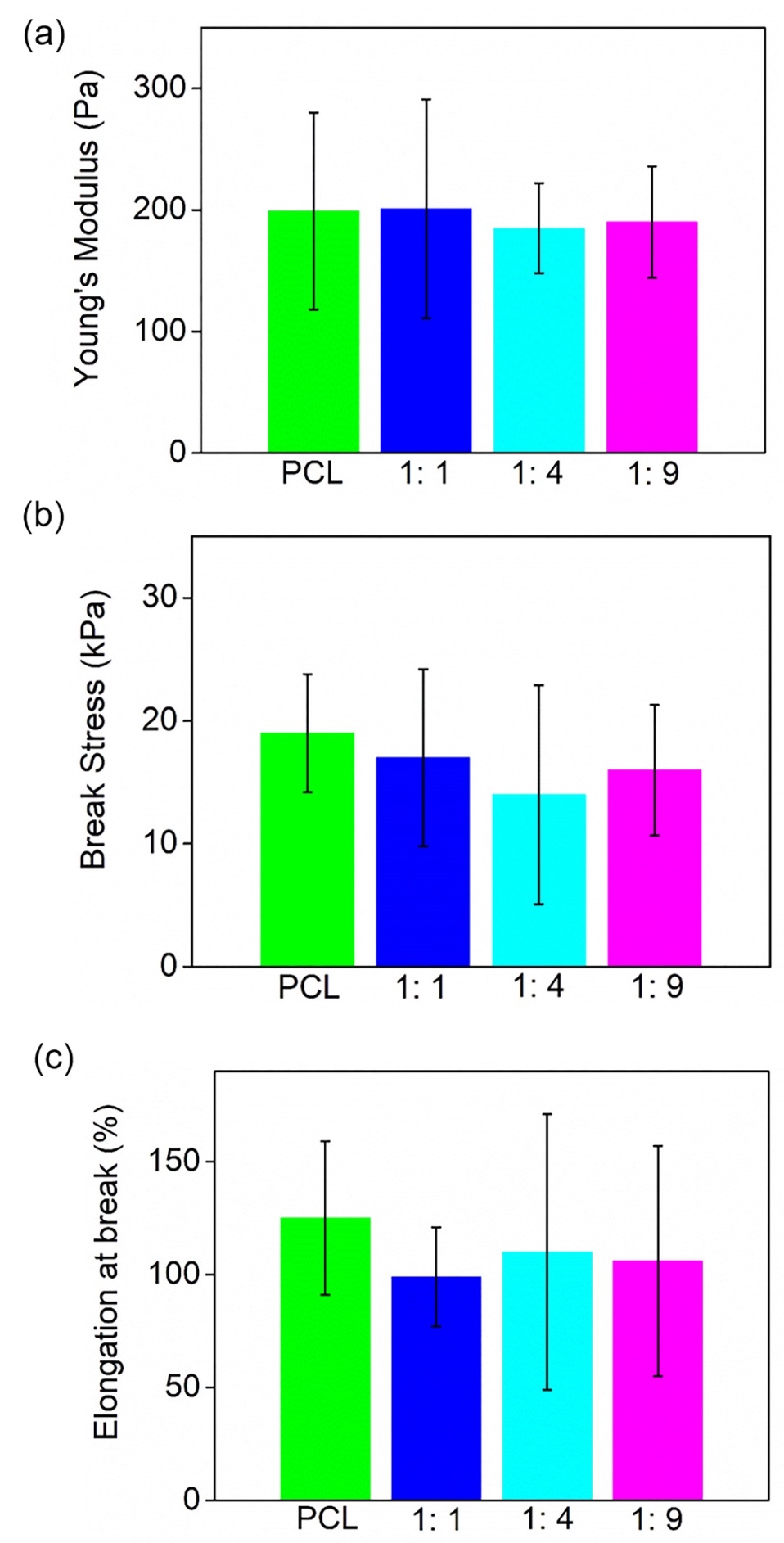


**Figure S2.** a) Young’s modulus at break of PCL and pMPC-grafted nanofibers. b) Break stress at break of PCL and pMPC-grafted nanofibers. c) Elongation at break of PCL and pMPC-grafted nanofibers.


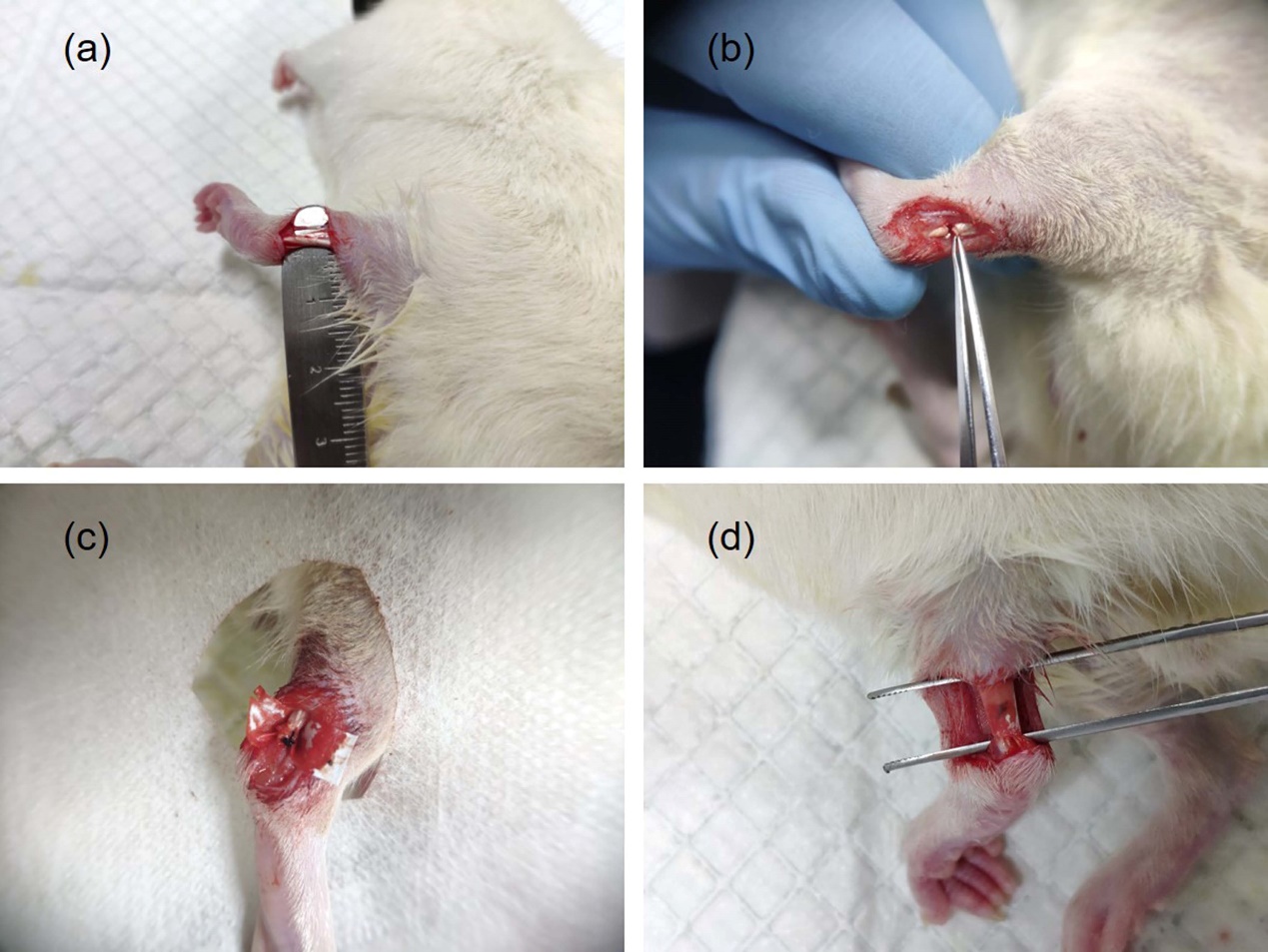


**Figure S3.** ***Operation process of rat Achilles tendon anti-adhesion.*** a) Exposure of the Achilles tendon in the limb. b) Transection of the tendon at the site of 5 mm away from the calcaneal tuberosity. c) Suture of the broken ends of the Achilles tendon in situ via modified Kessler technique and then prepare to wrap of the membranes around the repaired site. d) The view after wrapping of the membranes around the repaired site.
